# Long-term outcomes of external beam radiotherapy combined with high-dose-rate brachytherapy boost in intermediate- and high-risk prostate cancer

**DOI:** 10.3389/fonc.2026.1765212

**Published:** 2026-03-10

**Authors:** Kerem Tuna Tas, Tristan Spartmann, Edgar Smalec, Phillip Lishewski, Fatima Frosan Sheikhzadeh, Martin Boettcher, Klemens Zink, Ioanna Fragkandrea-Nixon, Johannes Huber, Ahmed Gawish, Sebastian Adeberg

**Affiliations:** 1 Department of Radiotherapy and Radiation Oncology, Philipps-Universität Marburg, Marburg, Germany; 2 Department of Radiotherapy and Radiation Oncology, Marburg University Hospital, Marburg, Germany; 3 Marburg Ion-Beam Therapy Center (MIT), Department of Radiotherapy and Radiation Oncology, Marburg University Hospital, Marburg, Germany; 4 University Cancer Center (UCT) Frankfurt – Marburg, Marburg, Germany; 5 The Beatson West of Scotland Cancer Centre, Glasogow, United Kingdom; 6 LOEWE Research Cluster for Advanced Medical Physics in Imaging and Therapy, (ADMIT), TH Mittel Hessen University of Applied Sciences, Giessen, Germany; 7 Department of Radiation oncology and Radiotherapy, University Hopspital Marburg, Marburg, Germany; 8 Department of Urology, Philipps-University Marburg, Marburg, Germany; 9 Department of Urology, Heidelberg University Hospital, Heidelberg, Germany

**Keywords:** androgen deprivation therapy, dose escalation, external beam radiotherapy, high-dose-rate brachytherapy, local control, progression-free survival, prostate cancer

## Abstract

**Background:**

For intermediate- and high-risk prostate cancer, dose escalation is essential to optimize oncological control. While external beam radiotherapy (EBRT) alone can be limited by dose constraints to adjacent organs-at-risk, high-dose-rate (HDR) brachytherapy provides a highly conformal boost option.

**Methods:**

This retrospective single-institution study analyzed 250 patients with localized intermediate- and high-risk prostate cancer treated between 06/2004 and 03/2024 with EBRT plus HDR brachytherapy boost. The EBRT dose averaged 50.4 Gy (range: 45–64 Gy), followed by HDR boost in nearly all patients (98.8%) with two fractions of 9 Gy. Androgen deprivation therapy (ADT) was administered to 39.2% of patients (98/250). Primary outcomes included local control (LC), progression-free survival (PFS), and overall survival (OS).

**Results:**

After a median follow-up of 63.5 months (mean 70.4, range 3–231), oncological outcomes were excellent. LC rates were 99.6% at 3 years, 98.8% at 5 years, and 98.4% at 10 years. PFS was 98%, 96.8%, and 96% at 3, 5, and 10 years, respectively. OS reached 98.4% at 5 years and 96% at 10 years. During the 231-month follow-up, 8.4% of patients developed biochemical recurrence, whereas in-field progression was observed in only 1.6%. Patients receiving ADT achieved 100% LC across all timepoints. Patterns of failure were predominantly distant (lymph nodes and bone). Acute and late toxicity was predominantly mild. No acute Grade ≥3 genitourinary (GU) or gastrointestinal (GI) toxicity was observed. Late Grade ≥3 toxicity was rare (0.8%, limited to GU events), and no late Grade ≥3 GI toxicity occurred.

**Conclusions:**

The combination of EBRT and HDR brachytherapy boost yields outstanding long-term LC, PFS, and OS for intermediate- and high-risk prostate cancer, confirming this regimen as a highly effective treatment strategy. The dominant pattern of failure was distant, underscoring the need for optimized systemic therapy integration in high-risk patients.

## Introduction

Prostate cancer remains one of the most prevalent malignancies among men worldwide, representing a significant public health concern (1). For patients with intermediate- and high-risk localized disease, achieving optimal oncological control while minimizing toxicity is a primary therapeutic goal. Numerous randomized controlled trials and large-scale analyses have consistently demonstrated that dose-escalation is a critical factor for improving biochemical control and survival outcomes in these patient groups (2–4).

While modern external beam radiotherapy (EBRT) techniques, such as intensity-modulated radiotherapy (IMRT) and volumetric modulated arc therapy (VMAT), allow for precise dose delivery, the proximity of critical organs-at-risk (e.g., rectum, bladder) can still limit the maximum dose that can be safely delivered to the prostate (5).

Brachytherapy (BT), particularly high-dose-rate brachytherapy (HDR-BT), offers a solution to this challenge. It provides a highly conformal method of dose escalation, delivering a large radiation dose directly to the target volume with a rapid dose fall-off to surrounding healthy tissues, making it an ideal modality for a boost treatment in combination with EBRT (6, 7).

EBRT with an HDR-BT boost has emerged as a highly effective treatment strategy, showing superior long-term biochemical control and survival rates compared to EBRT alone in several institutional series and meta-analyses (7–9). However, continuous evaluation of institutional outcomes and prognostic factors remains essential to validate and refine local treatment protocols.

In parallel, advances in external beam radiotherapy—including focal dose escalation via simultaneous integrated boost (SIB) with IMRT/VMAT, MR-Linac–guided adaptive radiotherapy, and stereotactic body radiotherapy (SBRT)—enable highly conformal targeting of dominant intraprostatic lesions.

These techniques enable selective dose intensification to biologically defined sub-volumes while minimizing exposure to surrounding organs-at-risk, representing promising alternatives or complements to HDR-BT boost strategies in modern prostate cancer management (10–12). Nevertheless, long-term clinical outcome data for these focal EBRT-based boost approaches remain limited, and HDR-BT continues to serve as a well-established reference modality for prostate dose escalation.

Against this background, the purpose of the present study is to evaluate long-term oncological outcomes and toxicity following EBRT combined with HDR-BT boost in patients with intermediate- and high-risk localized prostate cancer. In addition, this study aims to identify prognostic factors associated with treatment outcomes, thereby contributing mature follow-up data and real-world institutional evidence to the existing HDR-BT literature in the context of evolving focal boost techniques.

## Materials and methods

### Study design and patient selection

This single-center, retrospective analysis included 250 consecutive patients with histologically proven prostate cancer treated with a combination of EBRT and HDR-BT boost between 06/2004 and 03/2024. The study was conducted in accordance with the Declaration of Helsinki and approved by the local institutional review board (IRB approval number: 24-149-RS), with a waiver for informed consent due to the retrospective nature of the analysis.

Inclusion criteria comprised patients with localized intermediate- or high-risk prostate cancer according to NCCN guidelines. Exclusion criteria were the presence of distant metastases at diagnosis, prior radical prostatectomy, or receipt of radiotherapy (RT) to the pelvic region.

### Treatment protocol

All patients received combined-modality RT. The standard regimen consisted of:

- External Beam Radiotherapy (EBRT): A mean total dose of 50.4 Gy (range: 45–64 Gy) was delivered in fractions of a mean 1.89 Gy (range: 1.5–2.5 Gy). Of the 250 patients, 56 (22.4%) were treated with 3D-conformal radiotherapy (3D-CRT), while the remaining 194 (77.6%) received modern techniques, including intensity-modulated radiotherapy (IMRT) or volumetric modulated arc therapy (VMAT).

For EBRT, the clinical target volume (CTV) was defined as the prostate ± seminal vesicles according to risk stratification. The planning target volume (PTV) was generated by applying a uniform margin of 5–7 mm around the CTV, with posterior margin reduction to 3–5 mm at the rectal interface.

Image-guided radiotherapy (IGRT) was routinely performed using daily kilovoltage cone-beam CT (CBCT) or orthogonal portal imaging, depending on treatment era, to ensure accurate target positioning.

Variations in fractionation regimens primarily reflected evolving institutional protocols over the long study period and were influenced by treatment technique, risk group, and normal tissue constraints rather than patient-specific biological selection.

**Figure 1:**
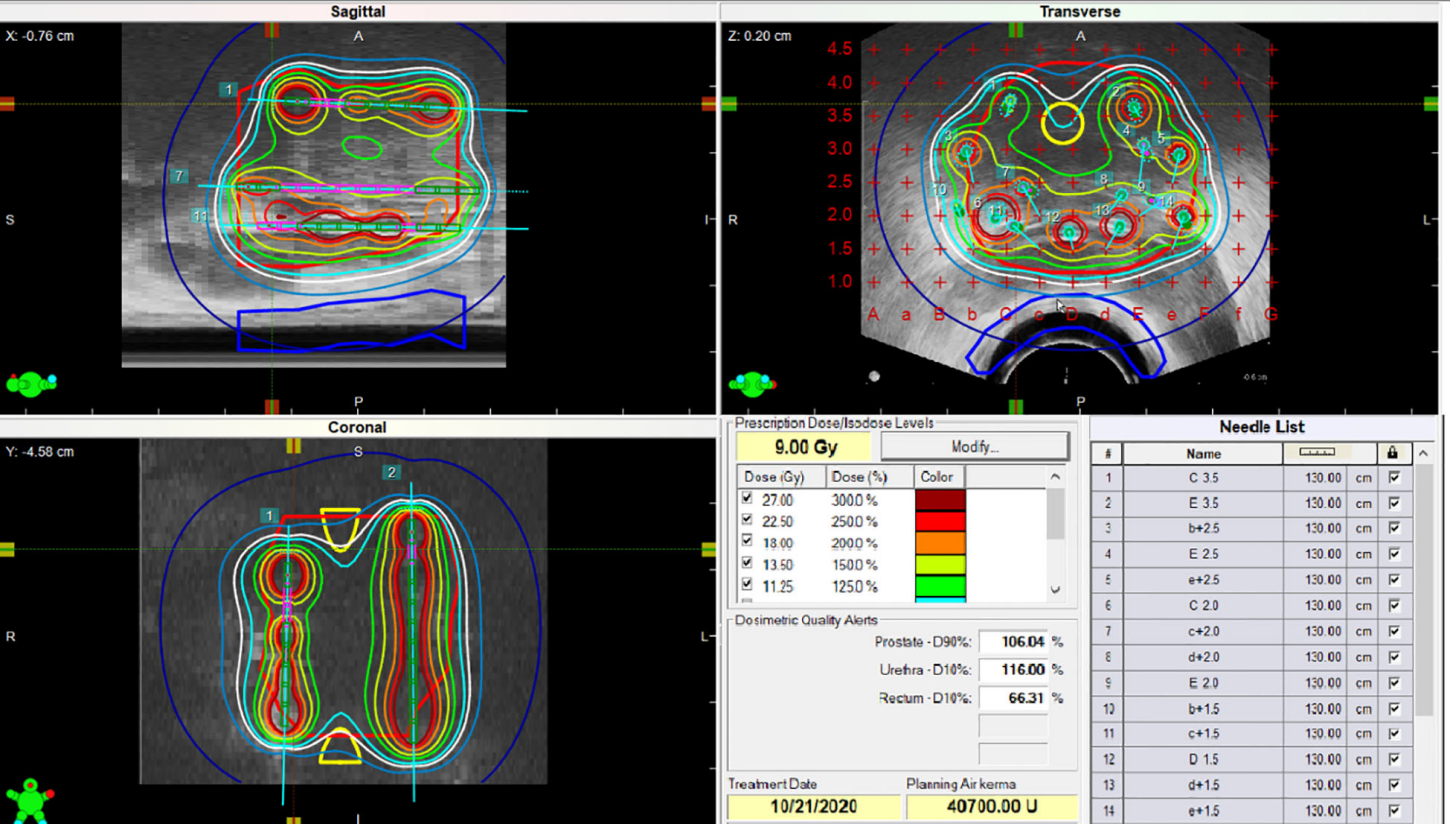
Representative HDR brachytherapy treatment plan for prostate cancer. The prescription dose was 9 Gy (100%). Target coverage demonstrates a prostate D90% of 106.4%, while organ-at-risk constraints are respected with a urethral D10% of 116.0% and a rectal D10% of 56.1%, all within predefined planning objectives.

- Brachytherapy Boost: Following or preceding EBRT, patients underwent HDR brachytherapy. The vast majority of patients (247, 98.8%) received two fractions of 9.0 Gy per fraction, while three patients (1.2%) received a single fraction (mean fraction dose 9.0 Gy; range: 9–14 Gy). Dose prescription and target definition followed international GEC-ESTRO and ABS recommendations, with the clinical target volume (CTV) defined as the prostate ± seminal vesicles. In accordance with these guidelines, no additional planning target volume (PTV) margin was applied. Treatment planning was based on intraoperative imaging for dosimetric optimization (13–16).
- Standard HDR brachytherapy dosimetric parameters were defined as follows: the prescription dose was 100% = 9 Gy. Acceptable target coverage was defined as a prostate D90% between 100% and 115% of the prescription dose. Organ-at-risk constraints included a urethral D10% ≤ 125% and a rectal D10% ≤ 75% of the prescription dose. A representative HDR treatment plan is illustrated in **Figure 1**.

Elective pelvic lymph node irradiation was not performed in this cohort. Radiotherapy was limited to the prostate ± seminal vesicles in all patients.

Androgen deprivation therapy (ADT) was administered at the discretion of the treating multidisciplinary team, primarily to patients with high-risk disease. In this cohort, 98 patients (39.2%) received ADT.

ADT use was defined as the administration of hormonal therapy for a minimum duration of ≥1 months. Among patients who received ADT, the median duration of therapy was 3 months (interquartile range [IQR]: 2–6 months; range: 1–36 months). When stratified by risk group, the median ADT duration was 3 months (IQR: 2.25–4.75 months) in intermediate-risk patients and 3 months (IQR: 2–6 months) in high-risk patients.

Detailed information on the specific type of ADT (e.g., LHRH agonist, LHRH antagonist, or combined androgen blockade) was not systematically captured in this retrospective dataset.

### Follow-up and outcome measures

Patients were followed regularly with clinical examination and prostate-specific antigen (PSA) measurements. The mean FU duration was 63.5 months (mean 70.4, range 3–231).

Patients with missing PSA values at specific follow-up time points were included in the analysis, and missing data were handled using a complete-case approach without imputation.

Initial PSA was defined as the PSA value at the time of diagnosis, whereas PSA before radiotherapy referred to the PSA level measured immediately prior to the start of EBRT.

The primary oncological outcomes were LC: Defined as the absence of clinically or radiologically detected recurrence within the prostate gland. PFS: Defined as freedom from biochemical failure (Phoenix definition: nadir + 2 ng/mL), local recurrence, regional lymph node recurrence, or distant metastasis. OS: Calculated from the start of treatment to death from any cause. Biochemical Failure: Assessed using the Phoenix definition (nadir + 2 ng/mL). Secondary outcomes included PSA nadir, the incidence of PSA bounce (a temporary rise in PSA of ≥0.2 ng/mL followed by a subsequent decrease to pre-bounce levels), and patterns of recurrence (in-field, lymph node, bone, other).

Treatment-related toxicity was assessed retrospectively based on clinical documentation during follow-up visits. Acute toxicity was defined as adverse events occurring within the first 3 months after radiotherapy, while late toxicity was defined as events occurring thereafter. Genitourinary (GU) and gastrointestinal (GI) toxicities were graded according to the Common Terminology Criteria for Adverse Events (CTCAE v5.0).

### Statistical analysis

Descriptive statistics were used to summarize patient, disease, and treatment characteristics. Continuous variables are presented as means with standard deviation (SD) or medians with range, as appropriate. Categorical variables are presented as frequencies and percentages.

Survival analyses for LC, PFS, and OS were performed using the Kaplan-Meier method. Univariate Cox proportional hazards regression models were used to assess the impact of various clinical variables (e.g., ADT use, age, T-stage, Gleason score, risk group, RT modality, total dose) on local control and progression-free survival. Results are reported as hazard ratios (HR) with corresponding 95% confidence intervals (CI) and p-values. A p-value of <0.05 was considered statistically significant.

All statistical analyses were performed using IBM SPSS Statistics (version 29; IBM Corp., Armonk, NY, USA).

## Results

### Patient characteristics

A total of 250 patients were included in this retrospective analysis. The cohort received combined percutaneous RT and BT. BT fraction dose (n=250): Mean 9.0 Gy (range 9–14 Gy, SD ±0.32); 247 patients received exactly 9 Gy. Number of BT fractions: 247 patients (98.8%) received 2 fractions, 3 patients (1.2%) received 1 fraction. Percutaneous RT total dose: Mean 50.4 Gy (range: 45–64 Gy, SD ±1.84). Percutaneous RT dose per fraction: Mean 1.89 Gy (range: 1.5–2.5 Gy, SD ±0.12). At the last FU, 234 patients (93.6%) were alive, while 16 patients (6.4%) had died. The median FU duration was 63.5 months (mean 70.4, range 3–231, SD ±55.2).

Baseline patient, disease, and treatment characteristics are summarized in **Table 1**. According to NCCN risk stratification, 181 patients (72.4%) had intermediate-risk disease, while 69 patients (27.6%) were classified as high risk.

**Table 1:** Characteristics of 250 patients and treatment.

Parameter	Value
Risk group (NCCN), n (%)	
Intermediate risk	181 (72.4)
High risk	69 (27.6)
Clinical T-stage, n (%)	
T1a	2 (0.8)
T1b	1 (0.4)
T1c	152 (60.8)
T2a	19 (7.6)
T2b	10 (4.0)
T2c	49 (19.6)
T3a	9 (3.6)
T3b	6 (2.4)
T3c	2 (0.8)
Gleason score, n (%)	
5	3 (1.2)
6	64 (25.6)
7a (3 + 4)	110 (44.0)
7b (4 + 3)	28 (11.2)
8	35 (14.0)
9	10 (4.0)
Initial PSA at diagnosis (ng/mL): Median (range)	7.8 (0.5–123.0)
PSA before radiotherapy (ng/mL): Median (range)	7.4 (0.01–123.0)
BT fraction dose, mean (range, SD)	9.0 Gy (9–14, ± 0.32)
Patients receiving exactly 9 Gy	247 (98.8%)
BT fraction number	1 fx: 3 (1.2%), 2 fx: 247 (98.8%)
Percutaneous dose, mean (range, SD)	50.4 Gy (45–64, ± 1.8)
Percutaneous dose per fraction, mean (range, SD)	1.89 Gy (1.5–2.5, ± 0.12)
Androgen deprivation therapy (ADT) use	98 (39.2%)
ADT use in high-risk patients	50/69 (72.5)
ADT use in intermediate-risk patients	48/181 (26.5)
ADT duration, mean ± SD (months)	1.8 ± 4.4 (range 0–36)
Pelvic lymph node irradiation	Not performed
Status at last follow-up	Alive: 234 (93.6%), Dead: 16 (6.4%)
Follow-up duration, mean (range, SD)	70 mo (3–231, ± 55)
Last PSA, median (mean, range, SD)	0.24 ng/mL (5.03, 0–436.5, ± 35)
PSA bounce	25 patients (10%)

BT, brachytherapy; FU, follow-up; PSA, prostate-specific antigen; mo, months; SD, standard deviation; ADT, androgen deprivation therapy.

The majority of patients presented with organ-confined disease, with 233 patients (93.2%) staged as T1–T2 and 17 patients (6.8%) as ≥T3.

Regarding tumor grade, most patients had Gleason score 7 disease (55.2%), while 18.0% presented with Gleason score ≥8.

Initial PSA values at diagnosis were available for 231 patients (92.4%), with a median PSA of 7.8 ng/mL (range 0.5–123.0). PSA values immediately prior to radiotherapy were available for 223 patients (89.2%), with a median PSA of 7.4 ng/mL (range 0.01–123.0).

### Treatment outcomes

Last PSA (n=156, missing=94): Median 0.24 ng/ml (mean 5.03, range 0–436.5, SD ±35.0). PSA bounce: Observed in 25 patients (10%) PFS: Mean 69 months (median 59.5, range 3–231, SD ±55). LC: Mean 70 months (median 63, range 3–231, SD ±54.7).

Biochemical failure: 21 patients (8.4%) experienced biochemical recurrence, while 229 (91.6%) remained recurrence-free. Recurrence sites: 11 patients with lymph node recurrence, 4 with in-field recurrence, 3 with bone recurrence, 1 with other organ involvement, 2 without further information. In-field progression (IFP): 4 patients (1.6%); 246 patients had no IFP.

The detailed treatment outcomes are summarized in **Table 2**.

**Table 2:** Treatment outcomes.

Parameter	Value
PFS, mean (median, range, SD)	69 mo (59.5, 3–231, ± 55)
LC, mean (median, range, SD)	70 mo (63, 3–231, ± 54.7)
Biochemical failure	Yes: 21 (8.4%), No: 229 (91.6%)
Recurrence sites	LN: 11, In-field: 4, Bone: 3, Other organ: 1, Unknown: 2
In-field progression	4 patients (1.6%)

PFS, Progression-free survival, LC, Local control, LN, Lymph node, SD, Standard deviation, mo, Months.

### Oncological outcomes

**Figure 2:**
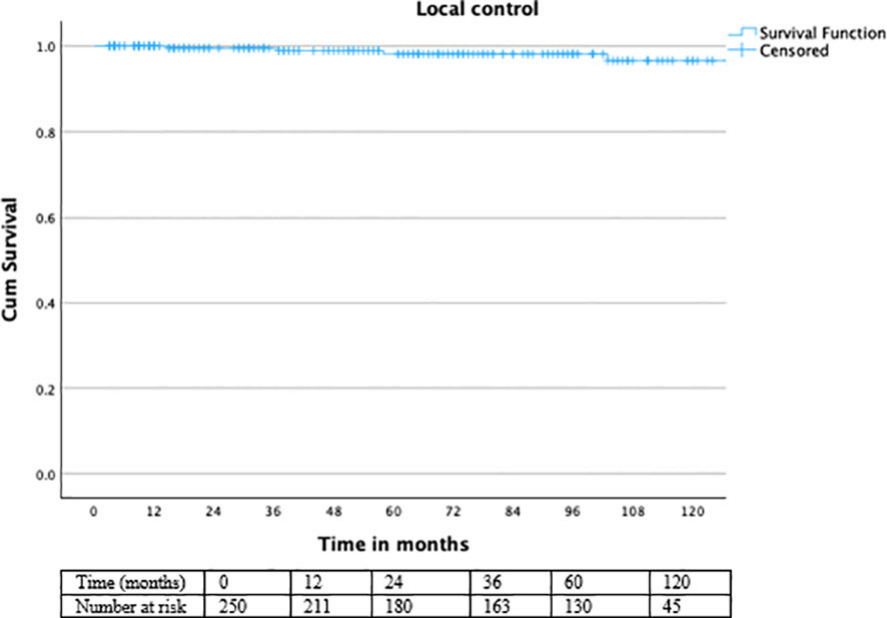
Kaplan–Meier curve for local control.

**Figure 3:**
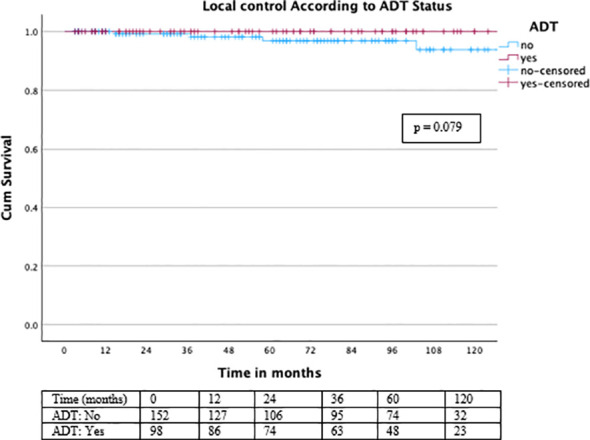
Local control stratified by ADT status. Kaplan–Meier analysis for LC stratified by ADT status demonstrated a trend toward improved LC in patients receiving ADT, although the difference did not reach statistical significance (log-rank χ² = 3.077, df = 1, p = 0.079).

In our cohort of 250 patients treated with a combination of BT and percutaneous RT, long-term LC, PFS, and OS outcomes were favorable. The 3-year LC rate was 99.6%, the 5-year LC rate was 98.8%, and the 10-year LC rate remained at 98.4%. When stratified by ADT, patients receiving ADT (n = 98) achieved 100% LC at all follow-up intervals. Kaplan–Meier analysis demonstrated excellent long-term local control overall (**Figure 2**), with consistently high rates maintained across ADT subgroups (**Figure 3**).

A trend toward improved LC was observed in patients receiving ADT; however, this difference did not reach statistical significance (log-rank χ² = 3.077, df = 1, p = 0.079).

Numbers at risk are shown below the Kaplan–Meier curves.

However, in our cohort, the duration of ADT varied substantially (1–36 months), which precluded a more differentiated subgroup analysis.

For PFS, the cohort showed rates of 98% at 3 years, 96.8% at 5 years, and 96% at 10 years.

**Figure 4:**
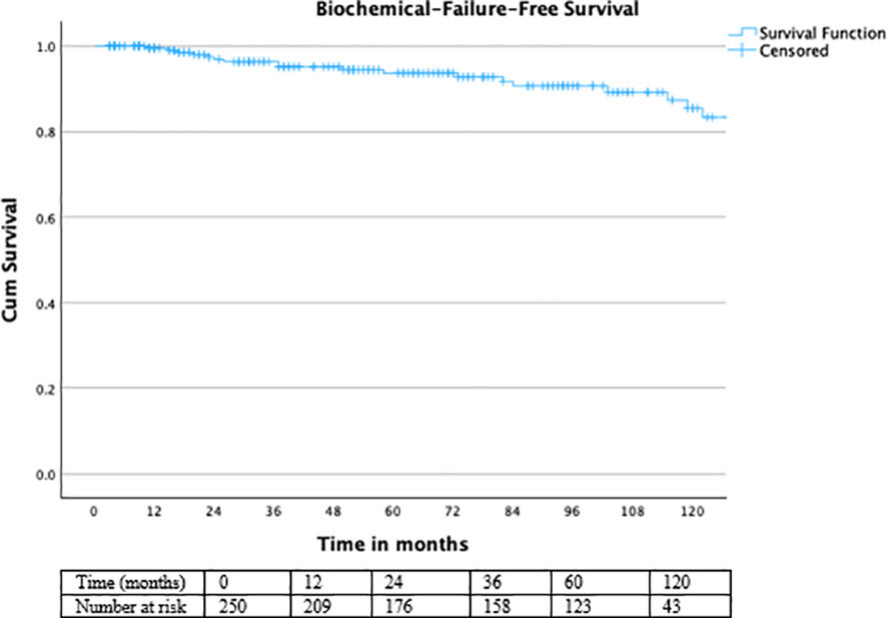
Kaplan–Meier curve for biochemical failure-free survival.

**Figure 5:**
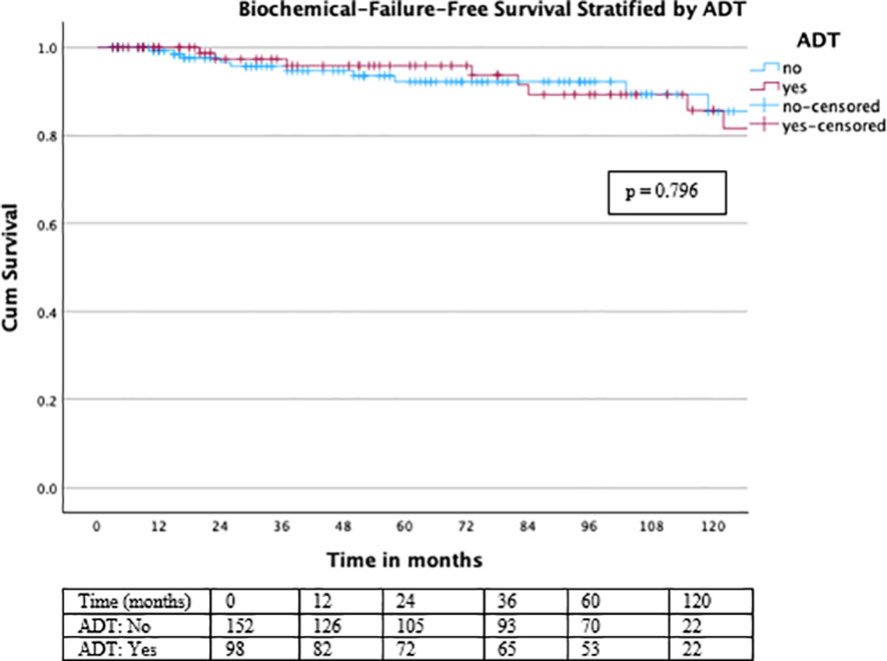
Biochemical failure -free survival stratified by ADT status. For biochemical failure–free survival, no significant difference was observed between patients treated with or without ADT (log-rank χ² = 0.067, df = 1, p = 0.796).

PFS remained favorable over long-term follow-up (**Figure 4**), with no significant difference between patients treated with or without ADT (log-rank χ² = 0.067, df = 1, p = 0.796) (**Figure 5**).

**Figure 6:**
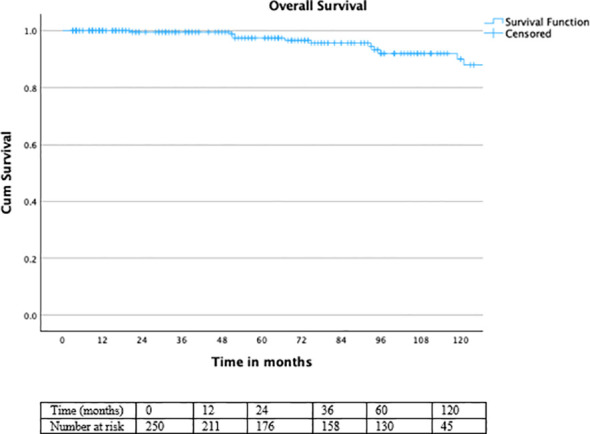
Kaplan–Meier curve for overall survival.

**Figure 7:**
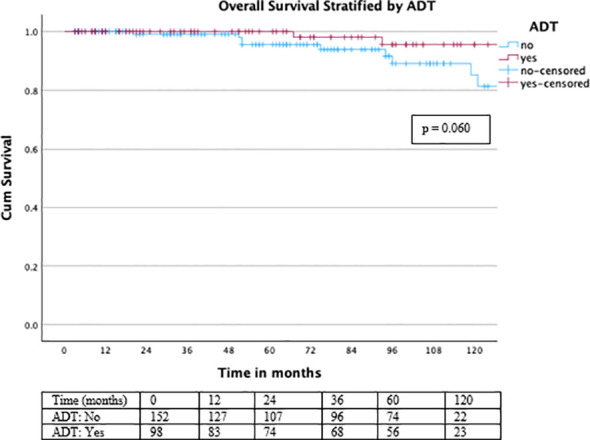
Overall survival stratified by ADT status. Overall survival analysis revealed a non-significant trend toward improved OS in the ADT group, without reaching statistical significance (log-rank χ² = 3.535, df = 1, p = 0.060).

Regarding OS, no deaths were observed within the first 3 years, while the 5-year OS rate was 98.4% and the 10-year OS rate was 96%. Overall survival was excellent throughout follow-up (**Figure 6**), with a non-significant trend toward improved OS in patients receiving ADT (log-rank χ² = 3.535, df = 1, p = 0.060) (**Figure 7**).

These results highlight the strong oncological effectiveness of combined BT and percutaneous RT in prostate cancer, particularly in patients receiving ADT, who demonstrated excellent long-term LC. The detailed oncological outcomes are summarized in **Table 3**. In addition, univariate Cox regression analyses were performed to evaluate potential prognostic factors for LC and PFS. None of the investigated variables, including ADT, PSMA-PET, age, T2 and upper stage, Gleason score, High risk group, RT modality, or total dose, showed a statistically significant association with LC (**Table 4**) or PFS (**Table 5**).

**Table 3:** Oncological outcomes (N = 250).

Timepoint	LC (%)	LC with ADT (%)	PFS (%)	OS (%)
3 years	99.6	100	98	100
5 years	98.8	100	96.8	98.4
10 years	98.4	100	96	96

LC, Local control, ADT, Androgen deprivation therapy, PFS, Progression-free survival, OS, Overall survival.

**Table 4:** Univariate Cox regression analysis for local control.

Variable	HR	p-value	95% CI (Lower–Upper)
ADT	0.02	0.343	0.00 – 67.3
PSMA-PET	6.94	0.095	0.71 – 67.5
Age >70	0.54	0.545	0.077 – 3.875
T2 ≥2	0.587	0.645	0.061 – 5.653
Gleason >7	1.88	0.591	0.191 – 18.38
High risk	0.97	0.981	0.101 – 9.38
RT modality (IMRT/VMAT vs. 3D-RT)	33.02	0.464	0.003 – 381268.2

HR, Hazard ratio, CI, Confidence interval, ADT, Androgen deprivation therapy, PSMA-PET, Prostate-specific membrane antigen positron emission tomography, T2, Tumor stage 2, RT, Radiotherapy, IMRT, Intensity-modulated radiotherapy, VMAT, Volumetric modulated arc therapy, 3D-RT, Three-dimensional conformal radiotherapy.

**Table 5:** Univariate Cox regression analysis for progression-free survival.

Variable	HR	p-value	95% CI (Lower–Upper)
ADT (yes vs no)	0.892	0.797	0.374 – 2.130
Age ≥70	0.722	0.461	0.304 – 1.715
T2 ≥2	0.934	0.887	0.366 – 2.386
Gleason >7	1.991	0.190	0.710 – 5.582
High Risk	0.950	0.921	0.347 – 2.604
RT modality (IMRT/VMAT vs 3D)	2.000	0.221	0.659 – 6.068
Total Dose >68 Gy	0.579	0.254	0.226 – 1.482

HR, Hazard ratio, CI, Confidence interval, ADT, Androgen deprivation therapy, T2, Tumor stage 2, RT, Radiotherapy, IMRT, Intensity-modulated radiotherapy, VMAT, Volumetric modulated arc therapy, 3D, Three-dimensional conformal radiotherapy, Gy, Gray.

### ADT distribution

ADT use differed markedly by risk group. Among high-risk patients, 50 of 69 (72.5%) received ADT, whereas only 48 of 181 intermediate-risk patients (26.5%) were treated with ADT.

### Treatment-related toxicity

Acute toxicity (≤3 months) was observed in 91 of 250 patients (36.4%) and was predominantly mild (CTCAE Grade 1). No acute Grade ≥3 GU or GI toxicity was recorded. Acute GU events included urinary incontinence in 6 patients (2.4%, all Grade 1), dysuria in 31 patients (12.4%; Grade 1: 27 [10.8%], Grade 2: 4 [1.6%]), nocturia/urinary urgency in 67 patients (26.8%; Grade 1: 62 [24.8%], Grade 2: 5 [2.0%]), and urinary retention in 7 patients (2.8%; Grade 1: 5 [2.0%], Grade 2: 2 [0.8%]). Acute GI toxicity was infrequent and limited to low-grade events: proctitis occurred in 6 patients (2.4%, all Grade 1) and rectal bleeding in 13 patients (5.2%; Grade 1: 11 [4.4%], Grade 2: 2 [0.8%]). Dermatitis occurred in 4 patients (1.6%, all Grade 1).

Late toxicity (>3 months) was documented in 90 of 250 patients (36.0%). Late GU toxicity remained predominantly Grade 1. Nocturia/urinary urgency was the most frequent late GU symptom, occurring in 76 patients (30.4%; Grade 1: 72 [28.8%], Grade 2: 4 [1.6%]). Dysuria was reported in 24 patients (9.6%; Grade 1: 22 [8.8%], Grade 2: 2 [0.8%]). Urinary incontinence occurred in 9 patients (3.6%, all Grade 1). Late urinary retention was observed in 8 patients (3.2%; Grade 1: 6 [2.4%], Grade 3: 2 [0.8%]). Late GI toxicity was rare: proctitis occurred in 3 patients (1.2%, all Grade 1) and rectal bleeding in 6 patients (2.4%; Grade 1: 5 [2.0%], Grade 2: 1 [0.4%]). No late Grade ≥3 GI toxicity was observed, and no dermatitis was recorded in the late period.

Detailed acute and late genitourinary and gastrointestinal toxicity profiles are summarized in **Table 6**.

**Table 6:** Acute and late treatment-related toxicity (CTCAE v5.0).

Acute toxicity (≤ 3 months)	Grade 1 n (%)	Grade 2 n (%)	Grade ≥3 n (%)	Total n (%)
Urinary incontinence	6 (2.4)	0 (0)	0 (0)	6 (2.4)
Dysuria	27 (10.8)	4 (1.6)	0 (0)	31 (12.4)
Nocturia/urinary urgency	62 (24.8)	5 (2.0)	0 (0)	67 (26.8)
Urinary retention	5 (2.0)	2 (0.8)	0 (0)	7 (2.8)
Proctitis	6 (2.4)	0 (0)	0 (0)	6 (2.4)
Rectal bleeding	11 (4.4)	2 (0.8)	0 (0)	13 (5.2)
Dermatitis	4 (1.6)	0 (0)	0 (0)	4 (1.6)
Late toxicity (> 3 months)				
Urinary incontinence	9 (3.6)	0 (0)	0 (0)	9 (3.6)
Dysuria	22 (8.8)	2 (0.8)	0 (0)	24 (9.6)
Nocturia/urinary urgency	72 (28.8)	4 (1.6)	0 (0)	76 (30.4)
Urinary retention	6 (2.4)	0 (0)	2 (0.8)	8 (3.2)
Proctitis	3 (1.2)	0 (0)	0 (0)	3 (1.2)
Rectal bleeding	5 (2.0)	1 (0.4)	0 (0)	6 (2.4)
Dermatitis	0 (0)	0 (0)	0 (0)	0 (0)

Acute toxicity was defined as adverse events occurring within the first 3 months after radiotherapy, and late toxicity as events occurring thereafter. Toxicities were graded according to the Common Terminology Criteria for Adverse Events (CTCAE). Percentages are calculated based on the total cohort (N = 250). No acute Grade ≥3 genitourinary or gastrointestinal toxicity was observed. Late Grade ≥3 toxicity was rare and limited to genitourinary events (0.8%).

## Discussion

This retrospective analysis of 250 patients with intermediate- and high-risk prostate cancer treated with a combination of EBRT and HDR-BT boost shows outstanding long-term oncological outcomes. Our findings, with a 10-year LC rate of 98.5%, a PFS rate of 93.6%, and an OS rate of 96.2%, strongly affirm the efficacy of this combined modality approach. The treatment regimen demonstrated excellent oncological efficacy, with a low rate of biochemical failure (8.4% at up to 231 months of FU) and a very low incidence of in-field progression (1.6%). In our single-institution cohort, data capture and FU were uniform, enabling consistent outcome ascertainment.

The paradigm of combining EBRT with a brachytherapy boost has been solidified by level one evidence. The pivotal ASCENDE-RT trial randomized patients to either dose-escalated EBRT (78 Gy) or EBRT (46 Gy) combined with a low-dose-rate (LDR) BT boost (115 Gy) (17). Their results demonstrated a significant advantage in biochemical failure-free survival for the BT boost arm, albeit with a different toxicity profile. Compared with historical brachytherapy-boost series, these low rates of high-grade late toxicity support the tolerability of HDR boost when delivered with standardized planning and adherence to organ-at-risk constraints.

While our study utilized HDR instead of LDR boost, the principle of profound dose escalation is the same. Our 10-year PFS of 93.6% compares favorably with the 9-year biochemical failure-free survival of 83% in the ASCENDE-RT BT boost arm. This is in line with the current German S3 guideline (Version 8.1, 2025), which recommends EBRT in combination with a brachytherapy boost (HDR or LDR) as a guideline-endorsed treatment option for patients with unfavorable intermediate- and high-risk prostate cancer. These guideline-concordant results support broad real-world applicability.

Our results are consistent with large institutional series and prospective studies of HDR boost. A multi-institutional pooled analysis by Ishiyama et al. (n=3,242) reported 5- and 10-year biochemical control rates of 90.6% and 81.4%, respectively (18). Similarly, Spratt et al. showed in a single-institution series that adding a BT boost improved biochemical control (92% vs 81%) and distant metastasis-free survival (97% vs 93%) compared with high-dose IMRT alone (19). Hoskin et al. (6) in a randomized trial demonstrated superior biochemical control with an HDR boost over EBRT alone; long-term MSKCC data further support durable control and survival with HDR boost (20). Our outcomes extend this evidence with decade-long estimates in a modern planning era.

The exceptionally high local control rates observed in our study (99.6% at 3 years, 98.9% at 5 years, and 98.5% at 10 years) are a cornerstone of this success. This suggests that the radical dose escalation achieved with the HDR boost is highly effective at eradicating localized disease within the prostate. Similarly, focal dose escalation with modern EBRT techniques, such as VMAT with a simultaneous integrated boost to the intraprostatic lesion, as demonstrated in the FLAME trial, has shown improved biochemical control without increasing toxicity (21, 22).

The pattern-of-failure analysis is particularly informative: among 21 biochemical failures, only 4 were confirmed in-field local recurrences, whereas the majority were nodal (n=11) or distant (e.g., bone, n=3) metastases.

It should be acknowledged that the long inclusion and follow-up period of this study spans several major paradigm shifts in prostate cancer management, including advances in radiotherapy delivery (3D-CRT to IMRT/VMAT), improvements in image guidance, the increasing use of PSMA-PET/CT for staging, and the evolution of systemic treatment strategies. These developments may have contributed to improved treatment precision and staging accuracy over time and could have influenced oncological outcomes, thereby limiting direct comparability between earlier and later treatment eras.

Accordingly, future protocols should prospectively integrate systemic strategies (e.g., optimized ADT duration, selective addition of novel hormonal agents) for patients at highest metastatic risk.

Elective pelvic lymph node irradiation was not part of the treatment protocol in this cohort. While randomized data such as the POP-RT trial have demonstrated improved biochemical progression-free survival with whole-pelvis radiotherapy in selected high-risk patients, the absence of pelvic irradiation in our study may have contributed to the predominance of nodal failures observed (23).

The rationale for omitting elective pelvic lymph node irradiation was based on the institutional treatment paradigm during much of the study period, which prioritized maximal intraprostatic dose escalation using HDR brachytherapy while minimizing treatment-related toxicity. During the earlier treatment eras included in this analysis, elective nodal irradiation in the absence of radiologically confirmed lymph node involvement was not uniformly adopted, despite estimated nodal risk based on tools such as the Roach formula. Furthermore, concerns regarding potential gastrointestinal toxicity and the lack of definitive survival benefit at the time influenced this treatment approach, even in patients with high-grade disease, including ISUP 5 tumors.

A key finding of our analysis was the impact of ADT on outcomes, particularly on LC. In our cohort, patients who received ADT (n=98) achieved a perfect 100% LC rate at all time intervals (3, 5, and 10 years). In contrast, the non-ADT group (n=152), while still achieving excellent results (97% at 10 years), showed a slight decline over time. This suggests a synergistic effect between ADT and radical RT. ADT is known to exert radiosensitizing effects by reducing tumor volume, inhibiting repopulation of clonogenic cells during radiotherapy, and potentially inducing apoptosis (24, 25). For high-risk disease, the combination of ADT with radiotherapy is the standard of care based on numerous trials (26, 27). Our data powerfully reinforce this principle, demonstrating that the combination of ADT with extreme dose escalation via HDR boost can virtually eliminate local recurrences.

As expected, ADT was preferentially administered to patients with high-risk disease, reflecting guideline-concordant clinical practice. This imbalance introduces inherent selection bias and limits the ability to isolate the independent effect of ADT on local control. In addition, the PSA-suppressive effect of ADT may partially mask early biochemical progression, which should be considered when interpreting PSA-based endpoints.

However, it is crucial to note that our univariate Cox regression analysis did not find ADT to be a statistically significant predictor for LC (HR = 0.92, p=0.338) or PFS (HR = 0.907, p=0.835). This lack of statistical significance is likely due to the very low number of events (local recurrences and progressions) in the entire cohort, which limits the statistical power to detect differences between subgroups. This is a common challenge in studies reporting exceptionally high success rates. The Kaplan–Meier curves for local control separate clearly, indicating a clinically meaningful benefit, although statistical power is limited. Future analyses with a larger sample size or a longer follow-up period to accumulate more events might be needed to confirm this relationship with statistical significance.

The treatment protocol in our study was highly standardized. The vast majority of patients (98.9%) received two fractions of 9.0 Gy HDR boost following EBRT to a mean dose of 50.4 Gy. This equates to a very high biological effective dose (BED), which is likely the fundamental driver behind the outstanding outcomes. The low standard deviations reported for both EBRT and BT doses (± 1.8 Gy and ±0.32 Gy, respectively) indicate excellent protocol adherence and quality assurance, which is critical in achieving consistent results in radiation oncology (6, 7, 28–30).

The analysis of PSA outcomes provides further insight into disease behavior post-treatment. The median last PSA of 0.24 ng/ml and the fact that the most frequent PSA nadir was ≤0.1 ng/ml are indicative of profound and durable treatment response. The rate of PSA bounce, observed in 10% of patients, is within the expected range reported in the literature (10-20%) and is a well-known benign phenomenon following brachytherapy that must be distinguished from true biochemical failure (31, 32). This distinction is crucial to avoid unnecessary interventions.

As previously mentioned, the pattern of failure is instructive. The dominance of lymph nodal and distant failures underscores a critical point: while combined EBRT/HDR boost is extremely effective at controlling disease within the prostate and seminal vesicles, patients with high-risk features remain at significant risk for micrometastatic disease present at the time of initial treatment. This highlights the importance of optimal systemic therapy and precise staging. The integration of advanced molecular imaging, such as PSMA-PET/CT, into initial staging could potentially upstage a portion of patients deemed to have localized disease by conventional imaging, thereby identifying those who might benefit from more aggressive or targeted systemic therapy (33, 34). In our univariate analysis, a positive PSMA-PET scan showed a strong trend toward being a predictor for worse LC (HR = 6.94, p=0.095), though it was not statistically significant, again likely due to low event rates.

The univariate Cox regression analyses for both LC and PFS revealed that none of the tested variables—including ADT use, age, T-stage, Gleason score >7, high-risk classification, RT modality, and total dose—reached statistical significance. This is a fascinating and important result. It suggests that within the context of this highly effective treatment regimen, the traditional risk stratification factors may lose some of their prognostic power. When local treatment is so effective, the differences in outcomes between, for example, a patient with Gleason 4 + 3 = 7 and a Gleason 4 + 4 = 8 may be minimized, as both are effectively treated within the prostate.

The very high HR for RT modality (IMRT/VMAT vs. 3D-CRT) is an artifact of the extremely small 3D-CRT cohort and the very low number of events, resulting in an unstable model. It should not be interpreted as IMRT being inferior; in fact, IMRT/VMAT are established standards due to superior normal tissue sparing and the possibility of safe dose escalation (5, 19, 34).

Our study’s outcomes align with the highest benchmarks in the literature, with 10-year LC, PFS, and OS rates confirming the effectiveness of your institutional protocol (6, 7, 35).

Our study’s regimen (EBRT: 50.4 Gy → HDR: 2x9 Gy) is a well-established and widely used fractionation scheme. It is biologically similar to other common regimens (e.g., 46 Gy + 2x10.5 Gy, 45 Gy + 2x9.5 Gy) and falls squarely within the range recommended by the GEC/ESTRO guidelines. The extremely high adherence to this protocol (low SD in doses, 98.8% receiving 2x9 Gy) is a key strength, as consistency is critical for achieving reproducible excellent outcomes (36).

Our finding that distant metastasis is the dominant mode of failure (11 lymph node, 3 bone) rather than local recurrence (4 in-field) is one of the most important observations and is entirely consistent with recent literature. As LC with dose-escalated RT has become exceedingly effective, the challenge in managing high-risk prostate cancer has shifted to controlling micrometastatic disease present at diagnosis. This is the central conclusion of large studies like Kishan et al. (37) and is a powerful argument for the integration of more effective systemic therapies (e.g., novel hormonal agents) for these patients.

Our findings align with and complement the existing body of evidence summarized in **Table 7**. This table highlights consistent improvements in biochemical and metastasis-free outcomes with HDR brachytherapy boost across prospective trials, meta-analyses, and guideline statements, reinforcing its role as a standard of care in unfavorable intermediate- and high-risk prostate cancer.

**Table 7:** HDR brachytherapy boost in localized prostate cancer – evidence summary.

Study (year)	Design	N	EBRT dose	HDR boost dose	Key oncological outcomes	Key conclusions & notes
Current Manuscript (2024)	Retrospective, single-institution	250	50.4 Gy (mean)	2×9 Gy (≈98.8%)	10-yr: LC 98.4%, PFS 96%, OS 96%; BF 8.4%; IFP 1.6%	Exceptional long−term control; failures predominantly distant.
Hoskin et al., Radiother Oncol (2012) (6)	Phase III randomized (EBRT vs EBRT+HDR)	218	55 Gy/20 fx (EBRT arm) vs 35.75 Gy/13 fx (combo arm)	2×8.5 Gy (within 24 h)	12-yr: median time to relapse 137 vs 82 months; ~21% absolute RFS benefit	Level−1 evidence: HDR boost improves biochemical control vs EBRT alone.
Henry et al., *Radiother Oncol* (2022) (36)	Expert guideline	—	Typically 40–50 Gy EBRT	Accepted regimens: 1×15 Gy, or 2×9.5–11 Gy	Summarizes evidence for BT boost superiority over EBRT alone	Recommends BT boost as a standard option for UIR/high−risk disease.
Hsu et al., Int J Radiat Oncol Biol Phys (2021) (38).	Prospective Phase II, multi-institutional	129	45 Gy/25 fx	19 Gy/2 fx (within 24 h)	BF: 14% (5y), 23% (10y); ≥G3 GU/GI ~4–5% at 5y	Excellent long−term control and acceptable toxicity in cooperative−group setting.
Chen et al., J Radiat Oncol Biol Phys (2021) (39).	Single-institution, PS-matched SBRT vs HDR	232 (131 SBRT; 101 HDR)	Pelvic EBRT (institutional)	HDR: 19 Gy/2 fx; SBRT: 19–21 Gy/2 fx	Similar biochemical control and MFS; modest toxicity	Suggests SBRT boost approximates HDR outcomes; prospective validation needed.
Joseph et al., Radiother Oncol (2016) (40)	Single-center prospective cohort	95	37.5 Gy/15 fx	Single 12.5 Gy	5-yr bDFS 80.5% (IR/HR)	Supports feasibility of single−fraction HDR boost + EBRT.
Morton GC et al., Clin Oncol (2020) (41)	Expert review	—	Various	Various	Summarizes RCTs/cohorts: BT boost improves bPFS vs EBRT alone	Argues BT boost should be considered standard for high−risk disease.
Novikov et al., Radiat Oncol J (2022) (42)	Retrospective HDR vs SBRT boost	149	3×7 Gy	2×10 Gy or 1×15 Gy	3- & 5-yr BRFS similar - 66–67%	Suggests SBRT boost may be non−inferior; further trials needed.
Patel SA et al., Brachytherapy (2025) (43)	ABS narrative review	—	45–50.4 Gy/25 fx	1x 15 Gy or 2 x 9.5–11 Gy	Synthesises recent data	Consensus: HDR−BT boost safe, effective, often preferable for dose−escalation.

EBRT, External Beam Radiotherapy; HDR, High-Dose-Rate (Brachytherapy); BT, Brachytherapy; UIR, Unfavorable Intermediate Risk; HR, High Risk; LC, Local Control; PFS, Progression-Free Survival; OS, Overall Survival; BF, Biochemical Failure; IFP, In-Field Progression; RFS, Relapse-Free Survival; bDFS, Biochemical Disease-Free Survival; bRFS, Biochemical Recurrence-Free Survival; MFS, Metastasis-Free Survival; BRFS, Biochemical Recurrence-Free Survival; GU, Genitourinary; GI, Gastrointestinal; ABS, American Brachytherapy Society; PS-matched, Propensity Score-matched; fx, fractions.

This study has several limitations inherent to its retrospective design. Firstly, the assignment of ADT was not randomized and was at the clinicians’ discretion, introducing potential selection bias. Patients receiving ADT likely had higher-risk disease, which would bias the results against showing a benefit for ADT. The fact that we still observed a strong clinical benefit for ADT in LC despite this potential bias is noteworthy. Secondly, as previously noted, the excellent outcomes and consequently low number of events limit the statistical power of the prognostic factor analysis.

Additional limitations include the lack of detailed information regarding the specific type of ADT administered and the absence of elective pelvic lymph node irradiation, which may limit direct comparison with more contemporary treatment strategies.

Importantly, no acute Grade ≥3 GU/GI toxicity was observed, and late Grade ≥3 toxicity was rare (0.8%, limited to GU events), with no late Grade ≥3 GI toxicity.

Finally, being a single-institution experience, the generalizability of our results may be influenced by the specific expertise and protocols of our center.

## Conclusion

In conclusion, this large retrospective analysis suggests that the combination of EBRT and HDR brachytherapy boost is associated with favorable long-term outcomes in patients with intermediate- and high-risk prostate cancer, including high rates of local control, progression-free survival, and overall survival with a favorable toxicity profile. The treatment regimen was delivered with high precision and consistency, as reflected by the low variability in dose parameters. Within the limitations of a retrospective, non-comparative design and low event rates, these findings support the feasibility and safety of EBRT combined with HDR brachytherapy boost as a treatment option for appropriately selected patients with localized prostate cancer who seek a definitive, dose-escalated radiotherapeutic approach.

## Data Availability

The raw data supporting the conclusions of this article will be made available by the authors, without undue reservation.
